# Detection of Antibodies Against *Leptospira interrogans* Serovars Among Stabled Horses in Qazvin Province of Iran as a One‐Health Concern

**DOI:** 10.1002/vms3.70520

**Published:** 2025-07-28

**Authors:** Mohsen Imandar, Amir Javadi, Gholamreza Abdollahpour, Parisa Rahimi Siahkal Mahale, Alireza Qanbari, Mostafa Mirzaalimohammadi, Eshagh Taherkhani, Meysam Olfatifar, Farhad Nikkhahi, Aida Vafae Eslahi, Milad Badri

**Affiliations:** ^1^ Qazvin Province Directorate of Iran Veterinary Organization Qazvin Iran; ^2^ Department of Internal Medicine Faculty of Veterinary Medicine University of Tehran Tehran Iran; ^3^ Faculty of Veterinary Medicine Urmia Branch Islamic Azad University Urmia Iran; ^4^ Gastroenterology and Hepatology Diseases Research Center Qom University of Medical Sciences Qom Iran; ^5^ Medical Microbiology Research Center Qazvin University of Medical Sciences Qazvin Iran

**Keywords:** horses, *Leptospira* spp, leptospirosis, seroprevalence, zoonotic disease

## Abstract

Leptospirosis, a global zoonotic disease caused by pathogenic *Leptospira* spp., poses significant health risks to both animals and humans. This study aimed to assess the seroprevalence of antibodies against *Leptospira interrogans* serovars in stabled horses in Qazvin province, Northwest Iran. From January 2023 to April 2024, a total of 83 blood samples were collected from asymptomatic horses using the microscopic agglutination test (MAT). Results indicated a seroprevalence of 40.96% (95% CI: 31–52), with notable variations among regions: 38.89% (95% CI: 27–52) in Qazvin, 38.46% (95% CI: 18–64) in Alborz and 50% (95% CI: 28–72) in Takestan. The Hardjo serovar was most prevalent (21.68%, 95% CI: 14–32), followed by Icterohaemorrhagiae (13.25%, 95% CI: 7–22) and Canicola (7.22%, 95% CI: 3–15). Statistical analysis revealed significant associations between seropositivity and factors such as the presence of domestic animals and housing conditions. Notably, adult horses exhibited higher seropositivity compared to younger ones. This study highlights the potential role of horses as reservoirs for *Leptospira*, particularly the Hardjo serovar, suggesting a zoonotic risk to humans and underscoring the need for effective surveillance and control measures in equine populations.

## Graphical abstract

• Totally, 40.96% of the stabled horses in Qazvin province, Northwest Iran were positive for antibodies against different *Leptospira interrogans* serovars.

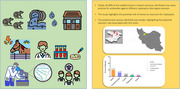

## 1 Introduction

Leptospirosis is the most widely distributed zoonotic disease globally, affecting every continent except Antarctica. Evidence of *Leptospira* has been discovered in nearly all mammal species studied (Badri et al. 2025; Dubey et al. 2021; Hamond et al. 2013). It is caused by gram‐negative spirochetes from the *Leptospira* genus, which is classified under the family Leptospiraceae and the order Spirochaetales (Pappas et al. 2008; Vijayachari et al. 2008). The disease was initially identified by physician Adolf Weil in Germany in 1886, who reported it as an ‘acute infectious condition characterized by spleen enlargement, jaundice and nephritis’ (Routray et al. 2018).

Humans typically acquire leptospirosis through contact with the urine of infected animals, either directly or through contaminated water or soil, whether in occupational, recreational or domestic settings. Leptospires are thin, spiral‐shaped bacteria, categorized into at least 12 pathogenic species and 4 saprophytic species, with more than 250 pathogenic serovars (Liu et al. 2024). Post‐infection immunity is generally, though not solely, provided by antibodies against leptospiral lipopolysaccharide (LPS) and is usually limited to serovars that are antigenically related (Adler and de la Peña Moctezuma 2010).

*Leptospira interrogans* infects a diverse range of animals, many of which show no symptoms despite being carriers of the bacteria in their kidneys. These animals release the bacteria into the environment through their urine and might not display symptoms until after an extended incubation period (Adler and de la Peña Moctezuma 2010). In horses, infections most commonly happen through direct contact with the urine or placenta of infected animals, or through indirect exposure to contaminated environments (Hamond et al. 2013).

*Leptospira* spp. infection in horses are often subclinical but are a major cause of abortions, stillbirths, weak foals and high neonatal mortality rates; when symptoms do occur, they typically include loss of appetite, mild fever, jaundice and frequent bouts of uveitis (Marzok et al. 2023). In pregnant horses, infections can cause placentitis, result in abortions or stillbirths, lead to neonatal mortality or result in the birth of weak foals (Hamond et al. 2014).

The classic icteric form of leptospirosis is typically seen in young animals but is rarely reported in adult horses. In addition, *Leptospira* spp. is recognized as a leading infectious cause of equine recurrent uveitis (ERU) and may also be associated with respiratory disorders (Verma et al. 2013; Broux et al. 2012; Hamond et al. 2012).

Diagnosing leptospirosis can be challenging and may require various methods, including antigen detection (PCR), serological testing, histological examination, bacterial culture and/or dark field microscopy (Khousheh et al. 2012). Serological methods are highly valuable for screening and occasionally diagnosing leptospirosis, particularly when assessing titer levels or comparing acute and convalescent samples. However, their effectiveness varies depending on the infection stage and the host's immune response. Although several techniques for detecting antibodies against *Leptospira* serovars have been developed, the microscopic agglutination test (MAT) with live antigens is the only method recognized by the World Organization for Animal Health (OIE) and is used as the standard reference method for both diagnosis and screening (Wasi'nski et al. 2021).

Epidemiological studies in horses typically use serological methods with various serovar panels as test antigens. The reported seroprevalence can range from 1% to more than 80%, depending on geographic location, the serovars evaluated, and differences in titer cut‐off values used in the studies (Hamond et al. 2013; Calderón et al. 2019; Ebani et al. 2012). The current study aimed to assess the prevalence of antibodies against *L. interrogans* serovars in stabled horses in Qazvin province, Northwest Iran.

## 2 Materials and Methods

### 2.1 Study Area

**FIGURE 1:**
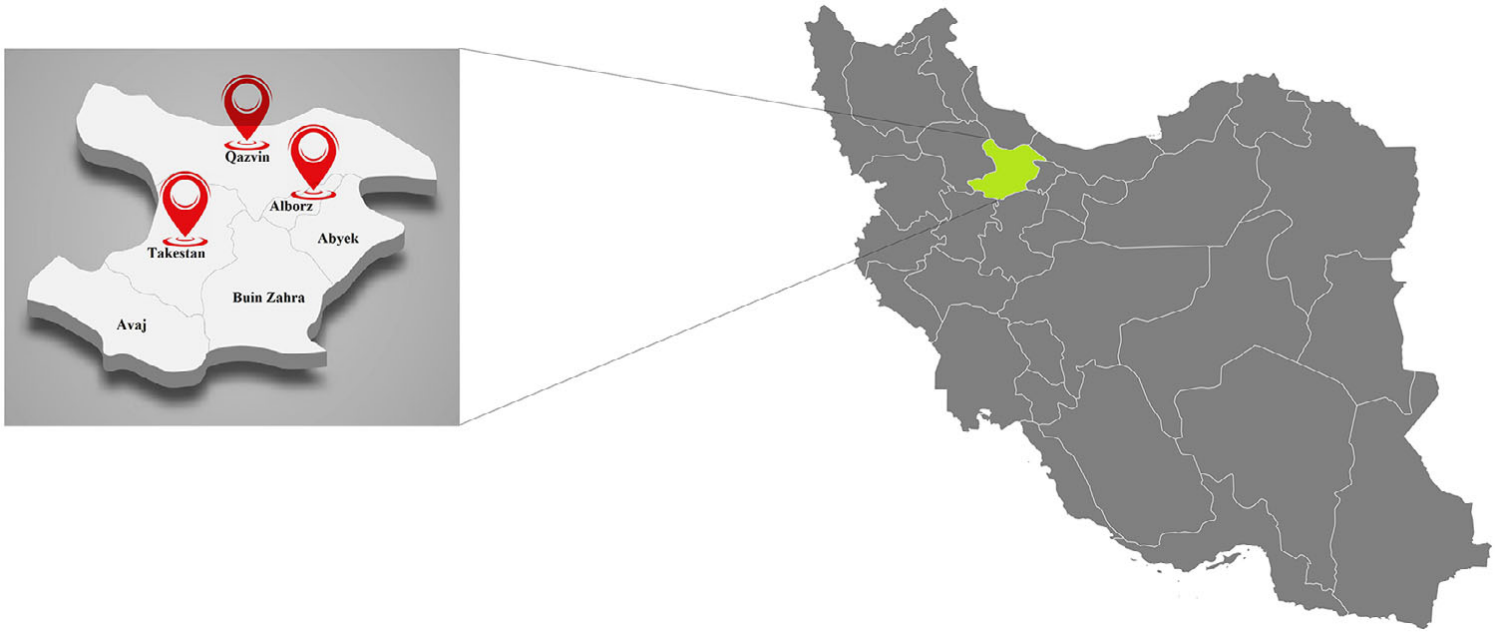
Map of Qazvin province, northwest of Iran, showing the sampling locations of horses included in the study. Red circles represent sampling sites.

From January 2023 to April 2024, a serological survey was carried out in Qazvin province, which spans 15,821 km^2^. This region lies between 48°45′ and 50°50′ east longitude and 35°37′ and 36°45′ north latitude, situated in the northwest part of Iran's central plateau. Qazvin province includes six counties: Abik, Avaj, Alborz, Buinzahra, Takestan, with Qazvin serving as the provincial capital (Figure 1) (Karyab et al. 2013).

Qazvin province is bordered to the north by Mazandaran and Guilan, to the west by Hamedan and Zanjan, to the south by Markazi and to the east by Tehran provinces (Mohammadi et al. 2025). The average annual precipitation in the region is approximately 280 mm, decreasing from north to south. The average temperature is 15.5°C, while potential evapotranspiration is around 2200 mm (Javadi et al. 2022).

### 2.2 Sampling and Epidemiological Feature

Eighty‐three blood samples were collected from horses in Qazvin province, with 54 samples from Qazvin city, 13 from Alborz and 16 from Takestan. All the sampled units relied on well water for their water supply, and the samples were gathered from mid‐autumn to late winter of the following year.

The horses examined were asymptomatic and had not been vaccinated against leptospirosis. In this study, no exclusion criteria were applied, nor was there any repeat sampling of the same horses. Blood samples were collected from the horses after obtaining informed consent from the farmers, using vacuum tubes to puncture the jugular vein without an anticoagulant. The samples were then centrifuged at 3000 × *g* for 10 min to separate the serum, which was stored at −20°C until serological analysis.

To identify potential risk factors associated with *Leptospira* seropositivity in horses, a structured questionnaire was administered to horse owners. The questionnaire collected data on various demographic and management‐related variables, including (1) horse characteristics: Sex and age (categorized as ≤3 years, 3–6 years, 6–9 years and ≥9 years); (2) breed: Arabian, Thoroughbred, Turkoman, KWPN, Holsteiner, Kurdish, Hanoverian and Trakehner; (3) rodent control practices: Presence or absence of a rodent control program; 4) animal exposure: Presence of other domestic animals and/or access to wild animals; (5) housing environment: Location of the horse (urban vs. rural) (Table S1).

### 2.3 Serological Analysis Procedures

**FIGURE 2:**
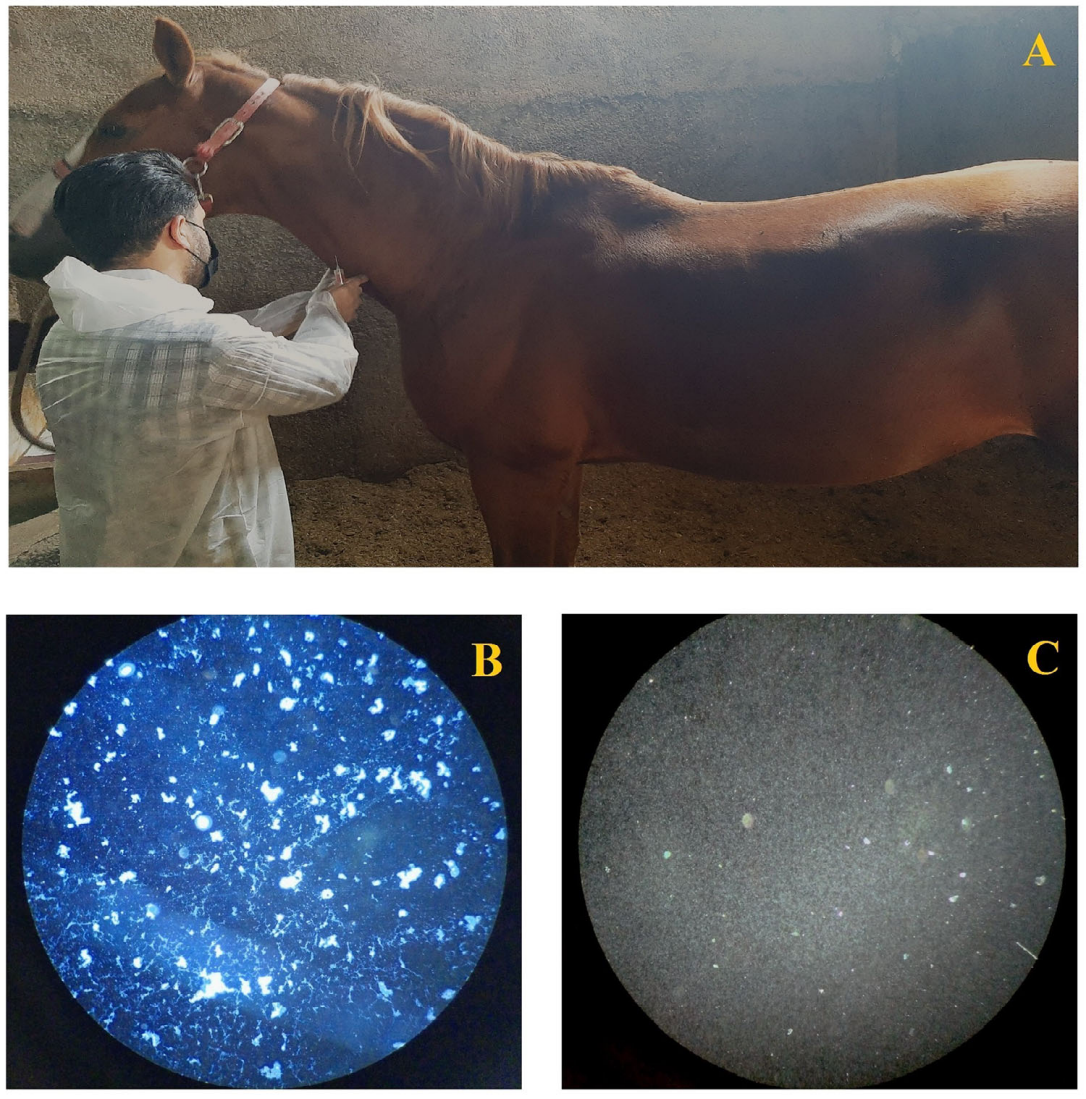
The blood samples were collected from the jugular vein of horses (A); Serum sample showing a positive case of *Leptospira serovar* by MAT test (B); Serum sample showing a negative case of *Leptospira* serovar by MAT test (C).

The MAT was performed to detect *Leptospira* antibodies in serum samples, following the procedures recommended by the OIE Terrestrial Manual (Chapter on Leptospirosis, 2021 edition) (World Organisation for Animal Health [OIE] 2021). All protocols were conducted at the *Leptospira* Research Laboratory, Faculty of Veterinary Medicine, University of Tehran, where reference strains of *L. interrogans* serovars (Hardjo, Icterohaemorrhagiae, Canicola, Grippotyphosa, and Pomona) were maintained in Ellinghausen–McCullough–Johnson–Harris (EMJH) medium and incubated at 28°C–30°C for 7–10 days until a concentration of approximately 2 × 10^8^ organisms/mL was reached, as confirmed by dark‐field microscopy (Figure 2).

The MAT procedure included positive control sera (with known titres against the tested serovars) and negative control sera (collected from unvaccinated horses with no history of exposure to *Leptospira* spp.). All serum samples were tested in duplicate to ensure reproducibility. Serum samples were initially screened at a 1:100 dilution by mixing 25 µL of serum, pre‐diluted 1:14 in sterile saline, with 150 µL of the standardized live antigen suspension. Samples showing positive agglutination were subsequently titrated in two‐fold serial dilutions, starting at 1:125, until the endpoint titre was established. A positive result was defined as ≥50% reduction in free leptospires at a titre of 1:100 or higher, compared to the negative control.

### 2.4 Statistical Analysis

Data were organized and analysed with SPSS software version 24 (IBM, Chicago, USA). The Chi‐square test was utilized to investigate the relationship between serology results (positive and negative) and various factors. A *p*‐value ≤0.05 was considered statistically significant.

### 2.5 Ethical Statement

The research protocol was approved by the ethics committee (code: IR.QUMS.REC.1403.301) at the Medical Microbiology Research Center of Qazvin University of Medical Sciences in Qazvin, Iran. The committee's animal ethics guidelines were strictly followed during the collection and handling of serum samples.

## 3 Results

Out of the total horses examined, 54 (65.06%) were female and 29 (34.93%) were male. The seroprevalence rate in males (41.37%, 95% CI: 26–59) was higher than in females (40.74%, 95% CI: 29–54). However, in the present study, no significant association was observed in either sex (*p* = 1.0) (Table 3).

Out of the total 83 serum samples examined, 34 (40.96%, 95% CI: 31–52) were positive for anti‐*Leptospira* antibodies. This includes 21 out of 54 samples from Qazvin (38.89%, 95% CI, 27–52), and 5 out of 13 from Alborz (38.46%, 95% CI: 18–64) with the highest prevalence observed in Takestan, where 8 out of 16 samples tested positive (50%, 95% CI: 28–72) (Table 3).

**FIGURE 3:**
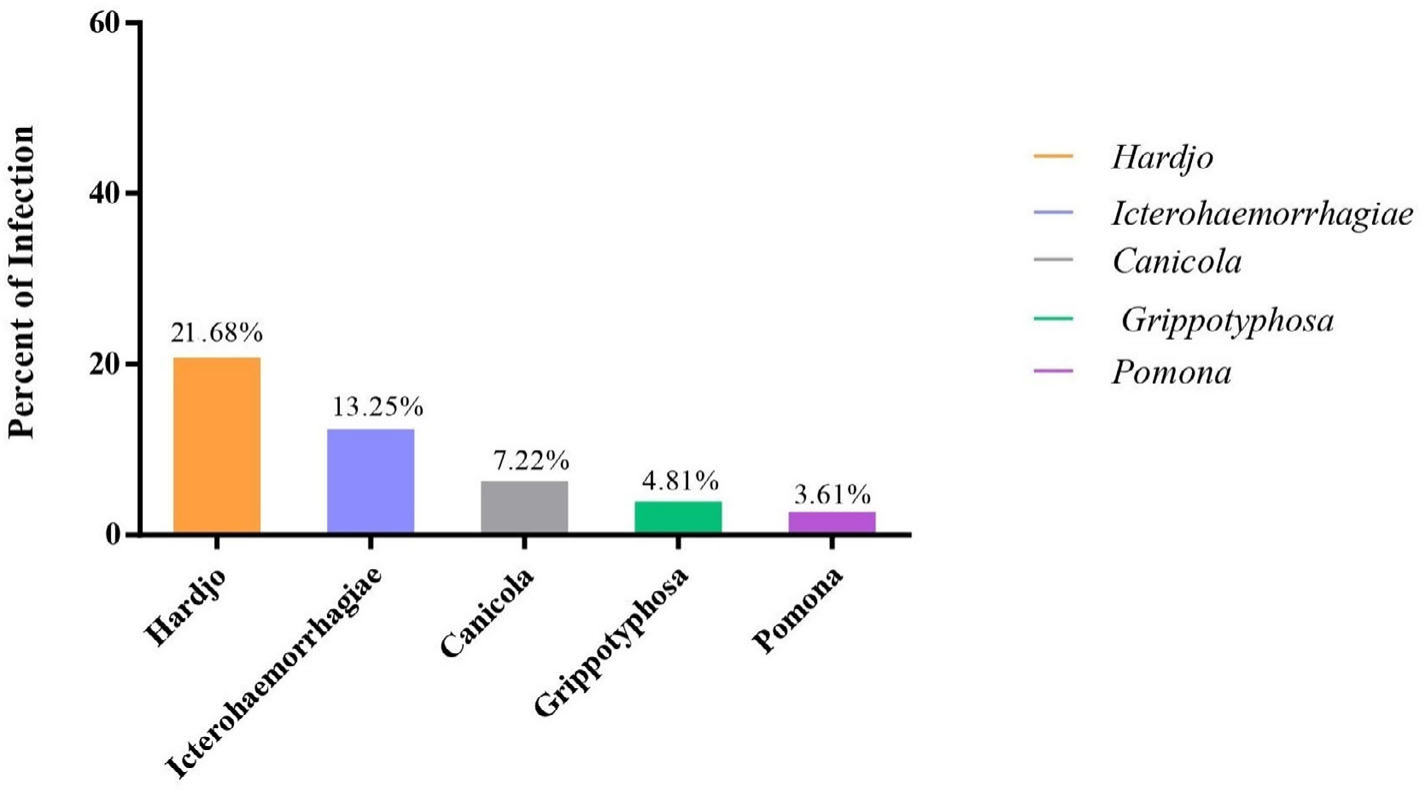
Bar chart showing antibody titers against *Leptospira interrogans* serovars in sampled horses. Titers are expressed as reciprocal serum dilution levels. Bars represent mean titers with standard deviation.

The positive antibody titres identified in this study were 1:100, 1:200, 1:400 and 1:1600. Each of the five serovars tested had at least one positive result, with Hardjo being the most common at 18 cases (21.68%, 95% CI: 14–32). The prevalence rates for the other serovars were as follows: Icterohaemorrhagiae at 11 (13.25%, 95% CI: 7–22), Canicola at 6 (7.22%, 95% CI: 3–15), Grippotyphosa at 4 (4.81%, 95% CI: 1–12) and Pomona at 3 (3.61%, 95% CI: 1–10) (Table 1 and Figure 3).

**TABLE 1:** Distribution of MAT antibody titers for each *Leptospira* serovar.

Types of serovar	NO. positive based on antibody titer				NO. positive/Total (%)	*p* value*
	1:100	1:200	1:400	1:1600		
Hardjo	10	7	1	0	18 (21.68)	0.0004
Icterohaemorrhagiae	7	3	1	0	11 (13.25)	
Canicola	4	1	0	1	6 (7.22)	
Grippotyphosa	3	1	0	0	4 (4.81)	
Pomona	3	0	0	0	3 (3.61)	

Among the total horses tested, 26 (31.32%, 95% CI: 22–42) were seropositive for a single serovar, while 8 (9.63%, 95% CI: 5–18) exhibited seropositivity for two serovars. The difference between these two groups was statistically significant (*p* < 0.001) (Table 2).

**TABLE 2:** Number of animals with single or two seropositivity.

NO. serovars	NO. positives (%)	*p* value*
1	26 (31.32)	0.001
2	8 (9.63)	

The age‐based analysis revealed that the highest rate of serovars was found in horses aged 9 years and older, with 13 out of 28 testing positive (46.43%, 95% CI: 30–64). In contrast, the lowest rate was recorded in the 3–6‐year age group, where 6 out of 19 tested positive (31.58%, 95% CI: 15–54). However, this difference was not statistically significant (*p* = 0.787) (Table 3).

**TABLE 3:** Distribution of seroprevalence for *Leptospira* spp. by age, region and management factors.

Risk factors	NO. examined	NO. positive	NO. negative	Seroprevalence and 95% CI	*p* value^*^
**Age**					
9 ≤	28	13	15	46.43% (95% CI: 30–64)	0.7871
6‐9	14	6	8	42.86% (95% CI: 21–67)	
3‐6	19	6	13	31.58% (95% CI: 15–54)	
3 ≥	22	9	13	40.91% (95% CI: 23–61)	
**County**					
Qazvin	54	21	33	38.89% (95% CI, 27–52)	0.7153
Alborz	13	5	8	38.46% (95% CI: 18–64)	
Takestan	16	8	8	50% (95% CI: 28–72)	
**Sex**					
Female	54	22	32	40.74% (95% CI: 29–54)	1.0
Male	29	12	17	41.37% (95% CI: 26–59)	
**Rodent control**					
Yes	25	8	17	32% (95% CI: 17–52)	0.397
No	58	26	32	44.83% (95% CI: 0.33–0.58)	
**Horse breeds**					
Arabian horse	15	1	14	6.66% (95% CI: 1–3)	0.0009995
Thoroughbred horse	11	2	9	18.18% (95% CI: 5–48)	
Turkoman horse	10	3	7	30% (95% CI: 11–6)	
Kwpn horse	39	24	15	61.54% (95% CI: 46–75)	
Holsteiner horse	2	1	1	50% (95% CI: 9–91)	
Kurdish horse	3	1	2	33.33% (95% CI: 6–79)	
Hanoverian horse	2	2	0	100% (95% CI: 34–100)	
Trakehner horse	1	0	0	0% (95% CI: 0–79)	
**Presence of other domestic animals** ^a^					
Cat	9	0	0	0% (95% CI: 0–30)	0.02399
Cat, Cattle, Dog	38	16	22	42.11% (95% CI: 28–58)	
Cat, Dog	36	18	18	50% (95% CI: 34–66)	
**Presence of wild animals** ^b^					
Fox	33	8	25	24.24% (95% CI: 13–41)	0.04099
Hedgehog	20	10	10	50% (95% CI: 30–70)	
Wild boars	30	16	14	53.33% (95% CI: 36–70)	

Abbreviation: CI, confidence interval.

^a^ Reported domestic animals (cats, dogs, and poultry).

^b^ Reported wild animals (foxes, Hedgehog and wild boars).

* In each case, *p*‐value estimated based on ch2 test between risk factors and positive and negative cases. Confidence Interval 95% (95% CIs) calculate via a website (https://www.statskingdom.com/proportion‐confidence‐interval‐calculator.html). A *p*‐value ≤0.05 was considered statistically significant.

The rodent control program on horse farms showed that the prevalence of *Leptospira* was significantly higher in farms lacking this program, at 44.83% (95% CI: 0.33–0.58). However, the differences were not statistically significant (*p* = 0.397) (Table 3).

Seroprevalence rates by horse breed indicated that Hanoverian horses accounted for the majority of seropositive cases (100%, 95% CI: 34–100). In addition, there was a highly significant statistical difference in the prevalence of *Leptospira* serovars among the various horse breeds (*p* < 0.0001) (Table 3).

In horse farms with other domestic animals, the highest rate of serovars was found in those with a regular presence of cats and dogs, at 50% (95% CI: 34–66). The most common serovars in these horses were Hardjo and Icterohaemorrhagiae, and the difference was statistically significant (*p* < 0.02) (Table 3).

In addition, the highest rate of serovars in horse farms with other wild animals was found in those that included wild boars, at 53.33% (95% CI: 36–70). The most common serovar in these horses was Hardjo, with a *p*‐value ≤ 0.05 considered statistically significant (Table 3).

According to the location of the farms, the majority of positive serovar cases were found in urban farms, with 16 out of 83 cases (19.27%, 95% CI: 12–29). Statistical analysis indicated a significant relationship between the prevalence of serovars in urban versus rural areas (*p* < 0.0001) (Table 4).

**TABLE 4:** Distribution of seroprevalence for *Leptospira* spp. based on the location of farm.

Horse club name (district)	Latitude	Longitude	Rural/Urban	Types of serovar	NO. positive/Total (%)	*p* value*
Club1 (Farm 1)	36.235136	50.036826	Urban	0	0	0.001
Club2 (Farm 2)	36.237437	50.044122	Urban	0	0	
Club 3 (Zoo, Village animal)	36.340548	50.063496	Urban	0	0	
Club 4 (Wasteland 1)	36.308151	49.962768	Rural	All serovars	8 (9.63)	
Club 5 (Wasteland 2)	36.280008	50.150265	Rural	3 serovars (Icterohaemorrhagiae, Hardjo, Canicola)	10 (12.04)	
Club 6 (Farm 3)	36.279572	49.962391	Urban	All serovars	16 (19.27)	

## 4 Discussion

Leptospirosis is a zoonotic disease caused by *Leptospira* spp. and is distributed worldwide. In Iran, the disease has re‐emerged in recent decades, with the first isolation of *Leptospira* from humans and cattle taking place in 1959 (Parhizgari et al. 2017). The disease is primarily found in low‐income populations within tropical developing countries (Picardeau 2015). It is estimated to result in more than 1 million severe cases and about 60,000 deaths each year. However, the shortcomings of surveillance systems in low‐income regions likely lead to an undercount of their actual impact. (Costa et al. 2015).

The current study examined the prevalence of antibodies against serovar‐specific *L. interrogans* antigens in stabled horses in Qazvin province using MAT (Wollanke et al. 2022). The prevalence of specific antibodies against the identified leptospiral serovars among stabled horses in this study (40.96%) is higher than that reported for native horses in various regions worldwide, such as New Zealand (24.8%) (Bolwell et al. 2020), Poland (15%) (Wasiński et al. 2021) and Ukraine (10.8%) (Ukhovskyi et al. 2023). In contrast, it is lower than the prevalence seen in horses from Mexico (97.1%) (Ramos‐Vázquez et al. 2024) and Brazil (44.46%) (de Sousa et al. 2020).

To date, the incidence and significance of leptospirosis in Iranian horses have not been well understood, and nearly all epidemiological studies have focused solely on serology. Consequently, the reported incidence has varied greatly depending on the geographical region studied (Verma et al. 2013; Arent et al. 2015). Horses are not usually regarded as a significant source of leptospirosis transmission compared to other livestock and wild animals. However, they can harbour leptospires in their kidneys, making them potential carriers and enabling the bacteria to spread in the environment (Hamond et al. 2014).

The overall prevalence of 40.96% among stabled horses in this study is nearly 2.5 times greater than the estimated prevalence of 19.99% (95% CI: 13.32–26.68%) for horses in Iran. The highest seropositivity for leptospirosis was recorded in East Azerbaijan at 41.05% (95% CI: 31.06–51.62), while the lowest was in Lorestan at 7.62% (95% CI: 3.35–14.46%) (Khalili et al. 2020).

Most research on animal leptospirosis in Iran has not explored the rodent control program or the presence of domestic animals in horse breeding stables. This study is the first in the country to tackle these important topics. A one‐health approach, which integrates the interactions between humans, animals and the environment, provides an ideal framework for understanding and addressing leptospirosis. Prevention and control strategies should be formulated within this context, but a major challenge has been the insufficient communication and collaboration between human and animal health professionals.

In endemic regions, titres of ≥ 800–1600, along with compatible symptoms, may be regarded as indicative of leptospirosis (Vera et al. 2019). None of the horses in this study exhibited clinical signs associated with leptospirosis. Asymptomatic leptospirosis in horses is significant because these animals are considered important in the transmission chain due to their close proximity to humans. Their often asymptomatic nature means they can act as potential silent reservoirs for the disease (Morais et al. 2024).

The findings of this study suggest that horses could serve as potential reservoirs for the Hardjo serovar, presenting a zoonotic risk for humans. This zoonotic threat should not be underestimated, and further studies are necessary to underscore the importance of the Hardjo serovar in terms of zoonotic transmission (Haggag et al. 2015).

Notably, a serological study of the West Central Iran tribal areas demonstrated that 54.1% of human seropositive samples reacted against serovar Hardjo, showing its predominance in the region's human infections (Ebrahimi et al. 2003). Also, ​a molecular study conducted in Iran identified *Leptospira* serovar Hardjo DNA in 9.78% of cattle (9 out of 92) and 4.85% of sheep (5 out of 103) blood samples, indicating the presence of this serovar among livestock populations (Khamesipour et al. 2014). These findings underscore the public health importance of serovar Hardjo and justify our focus on this serovar in the study.

In addition, the only serovar that exhibited 1:1600 antibody titres against *L. interrogans* was associated with the Canicola serovar. Dogs, which are typical reservoir hosts for common *Leptospira* serovars like Canicola, may have transmitted the infection to stabled horses through contact with infected dogs (Barmettler et al. 2011).

Our data reveal a significantly higher seropositivity to *L. interrogans* serovars in adult horses compared to younger ones. This observation is consistent with findings from other researchers (Blatti et al. 2011; Båverud et al. 2009; Rocha et al. 2004) and may be attributed to the increased likelihood of adults coming into contact with *Leptospira*. However, other researchers have reported no significant association between seropositivity and age (Wangdi et al. 2013). In this survey, there was no significant association between sex differences and seropositivity. Nonetheless, the study indicates that for Hanoverian horses, the risk of infection remains the same, regardless of how much time the animal spent grazing, turned out, or housed in the stable over the year.

The impact of housing type on the transmission of *Leptospira* spp. might be masked by the presence or absence of rodents, as they are a major risk factor for the prevalence of *Leptospira* (Hamond et al. 2014; Vera et al. 2019). Rodents are probably the main type of wildlife found in the indoor environments of horses, and a greater density of rats was positively associated with the prevalence of *L. interrogans*, which aligns with our findings. In stables lacking a rodent control program, the prevalence rate was higher, although the results did not show a statistically significant difference (Barwick et al. 1998).

The implementation of pest control measures significantly decreased the likelihood of Hanoverian horses contracting the infection. While assessing the role of rodents in the transmission of leptospiral serovars can sometimes be challenging (Simbizi et al. 2016), rodent control is recognized as a crucial factor for prevention (Hamond et al. 2014; Tsegay et al. 2016). In this study, the presence of other domestic animals statistically affected seroprevalence. The domestic animals included primarily dogs, cats and cattle. Their presence was not reported in only two farms, where the horses that had contact with cats living there tested negative for all examined serovars.

These results highlight the necessity for local public health authorities and veterinary clinicians to prioritize surveillance programs for stabled horses, especially in high‐prevalence regions. Vaccination campaigns against circulating serovars such as Hardjo and encouraging biosecurity practices (e.g., rodent control, domestic animal segregation) may reduce zoonotic transmission risks. Coordination between the veterinary and human health sectors within a One Health framework is essential to implementing integrated prevention strategies. We propose that Iran can increase its preparedness to zoonotic infections by: (1) Setting up a One Health coordination mechanism at the national level; (2) a shared zoonotic disease surveillance system; and (3) adopting international guidance and tools provided by World Health Organization (WHO), World Organisation for Animal Health (WOAH) and Food and Agriculture Organization of the United Nations (FAO).

### 4.1 Limitation

Our study faced the following limitations: (a) A larger sample size would provide more robust data and enhance the representativeness of different breeds, ages and locations of horses; (b) Our study focused on specific regions (Qazvin, Alborz, and Takestan), which may not reflect the prevalence of *Leptospira* in other areas. Regional environmental and ecological factors could significantly affect the transmission dynamics, necessitating broader geographic studies to fully understand the epidemiology of *Leptospira* in horse populations; (c) The seasonal variations were not considered in the current research. Leptospirosis can exhibit fluctuations based on environmental conditions such as rainfall and temperature, which affect bacterial survival and host behaviour. This limitation may have skewed our results, as higher rainfall is often associated with increased environmental contamination and exposure risk, potentially leading to under‐ or over‐estimation of infection rates depending on the timing of sample collection; (d) lack of molecular confirmation (e.g., PCR, culture) to detect active infection or carrier status.

Further studies must analyse environmental factors such as seasonal precipitation, temperature fluctuation and soil/water pollution levels, which may influence *Leptospira* survival and transmission dynamics. Expansion of the geographical location to include neighbouring provinces and longitudinal sampling across seasons would provide a more complete description of epidemiological trends.

## 5 Conclusion

Our study showed that the seropositivity rates differed by region, with Takestan representing the highest prevalence. The predominant serovar identified was Hardjo, highlighting the potential zoonotic risks associated with this strain. The findings underscore that age, with older horses showing higher seropositivity, may contribute to the likelihood of *Leptospira* exposure, while no significant association was found regarding sex. In addition, the presence of other domestic animals, particularly dogs and cats, significantly influenced seropositivity rates, suggesting that interspecies interactions could facilitate the spread of *Leptospira*. Although the absence of significant differences in rodent control programs indicates that further research is needed, the data suggest that improving rodent management in horse stables may be beneficial in reducing *Leptospira* transmission risk. The results emphasize the necessity of a One Health approach, integrating animal and human health strategies to mitigate the zoonotic threat posed by leptospirosis. This study not only contributes to the understanding of leptospirosis in horses but also calls for enhanced surveillance and control measures in equine populations to safeguard public health. Further research is warranted to explore the dynamics of *Leptospira* transmission in various environments and the role of horses as potential reservoirs in the context of zoonotic diseases.

## Author Contributions

**Mohsen Imandar**: conceptualization, investigation, methodology, supervision, project administration, data curation. **Amir Javadi**: investigation, validation, methodology, resources. **Gholamreza Abdollahpour**: investigation, conceptualization, resources. **Parisa Rahimi Siahkal Mahale**: investigation, validation, resources. **Alireza Qanbari**: investigation, validation, resources. **Mostafa Mirzaalimohammadi**: investigation, validation, resources. **Eshagh Taherkhani**: investigation, validation, resources. **Meysam Olfatifar**: formal analysis, software. **Farhad Nikkhahi**: conceptualization, investigation, methodology, supervision, data curation. **Aida Vafae Eslahi**: conceptualization, investigation, methodology, writing – review and editing, writing – original draft, resources, supervision, data curation, project administration. **Milad Badri**: conceptualization, investigation, funding acquisition, writing – original draft, writing ‐ review and editing, methodology, resources, supervision, data curation, visualization, project administration.

## Ethics Statement

The ethical approval was required and provided for this study, as stated by our institutional review board.

## Conflicts of Interest

The authors declared no conflicts of interest.

## Supporting information




**Supplementary File 1**: vms370520‐sup‐0001‐TableS1.xlsx

## Data Availability

The data that supports the findings of this study are available in the supplementary material of this article.
